# University of the City of New York

**Published:** 1851-12

**Authors:** 


*University of the City of New York.* — The medical department of this university have lately announced some changes in their system of instruction. The winter course of lectures is to be conducted, as heretofore, by the six professors constituting the Medical Faculty. On the 15th of March a second course of lectures begins, which is to be continued until the following October. Lectures during this summer term are to be given, in part, by the professors who lecture during the winter; but, in addition, a new corps of professors have been appointed, whose lectures are to be given solely during the summer. At the head of the list of the latter is the name of Prof. Chas. A. Lee, who is to lecture on medical jurisprudence. The other professors are as follows: B. W. Macready, on hygiene and toxicology: Thomas M. Markoe, pathological and microscopic anatomy; Wm. H. Van Buren, diseases of the genito-urinary organs; J. T. Metcalf, physical diagnosis and diseases of the chest; Dr. Bulkley is to give a course of lectures on diseases of the skin.

The faculty, in their announcement, state that this new plan has been adopted in order to enlarge the time and opportunities for medical instruction, and under the belief that students cannot be *coerced* to pursue a more extended course than that required by medical colleges generally. The advantages of the summer course are optional with the student, but it is proposed to confer on those who avail themselves of these advantages, certificates of honor.

We have not time, nor space, to comment on the above plan at this time. We will only remark that the university has been fortunate in having been able to secure the services of Prof. Lee. We may be allowed to say this, without intending any invidious reflections on the gentlemen associated with him.

